# Genome-wide analysis of enhancer RNA in gene regulation across 12 mouse tissues

**DOI:** 10.1038/srep12648

**Published:** 2015-07-29

**Authors:** Jen-Hao Cheng, David Zhi-Chao Pan, Zing Tsung-Yeh Tsai, Huai-Kuang Tsai

**Affiliations:** 1 Institute of Information Science, Academia Sinica, 128 Academia Road, Section 2, Nankang, Taipei 115, Taiwan

## Abstract

Enhancers play a crucial role in gene regulation but the participation of enhancer transcripts (i.e. enhancer RNA, eRNAs) in regulatory systems remains unclear. We provide a computational analysis on eRNAs using genome-wide data across 12 mouse tissues. The expression of genes targeted by transcribing enhancer is positively correlated with eRNA expression and significantly higher than expression of genes targeted by non-transcribing enhancers. This result implies eRNA transcription indicates a state of enhancer that further increases gene expression. This state of enhancer is tissue-specific, as the same enhancer differentially transcribes eRNAs across tissues. Therefore, the presence of eRNAs describes a tissue-specific state of enhancer that is generally associated with higher expressed target genes, surmising as to whether eRNAs have gene activation potential. We further found a large number of eRNAs contain regions in which sequences and secondary structures are similar to microRNAs. Interestingly, an increasing number of recent studies hypothesize that microRNAs may switch from their general repressive role to an activating role when targeting promoter sequences. Collectively, our results provide speculation that eRNAs may be associated with the selective activation of enhancer target genes.

Long-range interaction between enhancers and promoters is particularly crucial but involves a convoluted transcriptional mechanism. Enhancers are distal-acting elements that increase target gene expression even when residing millions of base pairs away^1^. Obscurity in the understanding of enhancer-regulated transcription arises due to the fact that enhancers selectively activate genes in a well-controlled tissue- and temporal-specific manner under various conditions and developmental stages^2^. Further complications are introduced by the recent discovery that widespread transcription occurs not only at promoters but also at enhancers^3^,^4^,^5^. Enhancer loci are found to recruit RNA polymerase II and express noncoding RNAs, known as enhancer RNAs (eRNAs). Current knowledge on eRNAs is far from being comprehensive. Kim *et al.* reveal that knocking out the target promoter of an enhancer subsequently abolishes eRNA transcription^4^. Large-scale analyses show the expression level of eRNAs is positively correlated with the expression level of nearby genes^4^,^6^ and target genes^7^ of the corresponding enhancer, and may be indirectly induced by transcription factors^4^,^8^,^9^,^10^,^11^,^12^,^13^. Several studies further suggest that eRNAs contribute to enhancer-regulated transcription^9^,^10^,^11^,^12^,^13^. These studies demonstrate that eRNA is closely associated with target genes of the corresponding enhancer, and thus entail a detailed examination of enhancer-regulated transcription with the incorporation of eRNAs.

However, not all enhancers possess eRNAs. According to Kim *et al.*‘s study^4^, only around half of the intergenic enhancers transcribe eRNAs. Other research similarly did not detect transcripts from a proportionate number of active enhancers^11^,^14^,^15^, although expression of eRNAs is positively correlated with H3K4ac27 levels, which is a marker for active enhancers^14^. This controversy continues, as one study shows inhibiting eRNA transcription does not affect enhancer-promoter looping^16^, while another reported that eRNAs are important for looping formation^10^. Summing these evidences suggest that while eRNA transcription may be highly dependent on enhancer-promoter interaction, the reverse may be untrue—some active enhancers might not necessarily couple with eRNA transcripts. It further implies that enhancers could possess two states as distinguished by the presence of eRNAs. Therefore it is of interest to investigate the differences in the expression and function of enhancer-regulated genes based on eRNA transcription.

The occurrence of eRNA transcription calls for investigations on whether the presence of eRNAs could be an indication of expressional differences in enhancer-regulated transcription. The positive correlation between expression level of eRNA and enhancer target genes immediately draw speculation on whether eRNAs could be a new type of positive regulators^3^. Indeed, several reports show that knocking down eRNA also reduces expression of nearby or target genes in various tissues and species^9^,^10^,^11^,^12^,^13^. Therefore, at least some cases demonstrate that eRNAs have a positive contribution to enhancer-regulated transcription. Additionally, an increasing number of studies have reported a new type of noncoding RNAs (ncRNAs) that play part in gene activation known as ncRNA activation (ncRNA-a)^17^,^18^,^19^. Another type of ncRNA that has been hypothesized to have activating ability is a subset of microRNAs (miRNAs), which facilitates transcription when targeting promoters even though miRNAs are generally considered as a repressor^20^,^21^,^22^,^23^,^24^,^25^. miRNAs activate gene expression through complementarily binding to the promoter sequences of target genes^20^,^21^. Though, it still remains unclear if those activating miRNAs and those specific eRNAs with positive regulatory contribution have any similar properties. There is also yet to be a genome-wide study that investigates if the eRNA-associated increase in expression is limited to specific cases or is a general phenomenon.

In this study, we *in silico* examined eRNAs in the enhancer-promoter relationship by analyzing genome-wide data on 12 mouse tissues. We reported that the enhancers transcribing eRNAs are globally consistent with a significantly higher expression and more tissue-specific functions in their target genes to those enhancers not-transcribing eRNAs, indicating presence of eRNA may distinguish enhancers into two states. The same enhancers across tissues may also transcribe eRNAs in some tissues but not in other tissues, reinforcing the two states and tissue-specificity of enhancers and eRNAs. Surprisingly, we further discovered that eRNAs contain regions similar to miRNA in sequence and secondary structure, and interestingly some complement regions in target promoters of the corresponding enhancer. Together, our results demonstrate that enhancers possess two states as distinguished by eRNAs, the presence of eRNA is related to a genome-wide and cross-tissue increase in target gene expression, and we provide a speculation that eRNAs add an additional layer of regulation in enhancer-regulated transcriptional control.

## Results and Discussion

### Prevalence of transcribing and non-transcribing enhancers indicate two states of enhancer-regulated transcription

**Figure 1:**
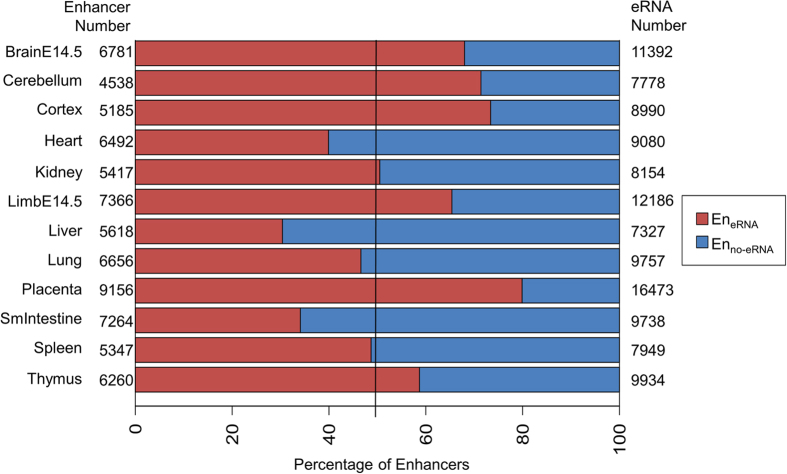
Proportions of number of En_eRNA_ and En_no-eRNA_. Red denotes the percentage of enhancers transcribing eRNAs. Blue denotes those not transcribing eRNAs. The black line represents the 50% cutoff. The number of enhancers with and without eRNAs is listed to the left of the bar graph. The number of eRNAs is listed to the right of the bar graph.

To investigate if eRNAs are globally associated with enhancer-regulated transcription, we first examined the prevalence of enhancer transcription. Although previous studies have highlighted that transcription at enhancers is widespread^6^,^14^, the existence of non-transcribing active enhancers^11^,^14^,^15^ are often neglected. We hence conducted a genome-wide and cross-tissue analysis to determine enhancers that transcribe eRNAs. The enhancers of 12 tissues were obtained from Shen *et al.*^26^. Shen *et al.* identified enhancers by signals of histone markers and coactivators, and enhancer target genes by correlating the signals of histone markers and RNA polymerase II. A subset of their data is verified with luciferase assays, 3C and Hi-C experiments. The eRNAs are RNA-seq contigs obtained from ENCODE^14^, where they assembled contigs from contiguous regions covered by uniquely aligned reads. Following the eRNA determination method by ENCODE^14^, intergenic enhancers that contain the 5′ start of a RNA-seq contig are considered as an enhancer transcribing eRNA (En_eRNA_) and the contig as an eRNA. Conversely, an enhancer not-transcribing eRNA (En_no-eRNA_) is defined as an intergenic enhancers without a RNA-seq contig. Note that En_no-eRNA_ do not refer to transcriptionally silent enhancers, and these regions may contain lowly expressed RNA transcripts that could not be assembled or are undetectable by current technology. We identified between 2000 to 4000 En_eRNA_ for each of the 12 tissues. Among the examined tissues, at least 1/3 (1709 En_eRNA_, liver) of the intergenic enhancers transcribe eRNAs (Fig. 1). Conversely, at least 1/5 (7317 En_no-eRNA_, placenta) of the enhancers do not transcribe eRNAs. The results verify that transcription at enhancers is indeed prevalent, but non-transcribing enhancers still exist in non-negligible proportions. Even with numerous experimental confirmations to support these enhancers are bona fide^26^, we still discovered substantial portions of these enhancers do not transcribe eRNAs. A likely explanation for this discovery is that enhancers might exist in two states as distinguished by the presence of eRNAs. It hence draws attention to whether the presence of eRNAs at enhancers would correspond to any transcriptional differences in enhancer-regulated transcription.

### Presence of eRNA corresponds with a higher expression level in target genes

**Figure 2:**
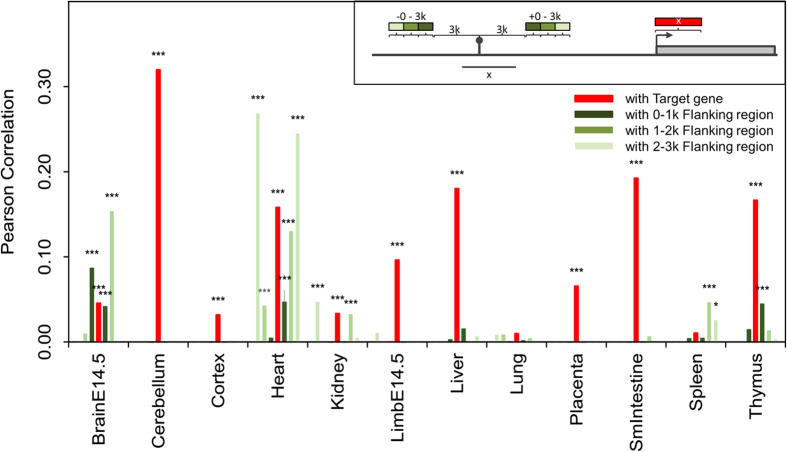
Correlation of expression level of eRNAs with the enhancer’s target gene or flanking regions. Each bar represents the Pearson correlation between the expression level of eRNA and target gene (red), eRNA and 0–1 k flanking region (dark green), eRNA and 1–2 k flanking region (green), or eRNA and 2–3 k flanking region (light green). The expression level is calculated by BPKM. Significance is denoted by ^*^p-value < 0.001; ^***^p-value < 10^−5^.

To further investigate the possibility of eRNAs differentiating enhancers into two states, we examined if there are differences in the expression level of their target genes. Although previous studies have shown that expression level of eRNAs is positively correlated to the expression level of nearby or target genes of the corresponding enhancer^4^,^6^,^7^, these studies did not consider potential biases resulting from regions highly transcribed by RNA polymerase II. To eliminate this possibility, we calculated the expression correlation between eRNAs and their corresponding target genes, and then compared this correlation to the expression correlation between eRNAs and three background regions: enhancer flanking regions, gene upstream regions, and random intergenic regions. Expression level of eRNA is positively correlated with that of target genes in almost all the tissues (Fig. 2). For the most part, the correlations of enhancer flanking regions are insignificant and lower than the correlation of eRNAs. Moreover, the correlation of eRNA expression level with gene upstream regions and random intergenic regions is low and insignificant in all examined tissues (Table S1). We therefore show that the positive and significant correlation between eRNAs and target genes is unaffected by highly transcribed regions, as demonstrated by comparing with background regions.

**Figure 3:**
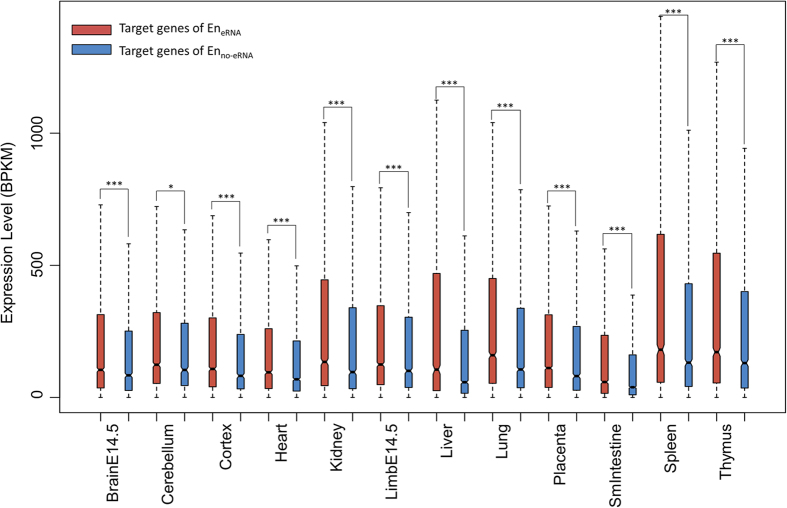
Expression level of target genes of En_eRNA_ and of En_no-eRNA_. Each box represents the distribution of the BPKM of the target genes of En_eRNA_ and of En_no-eRNA_. Significance is determined by a one-sided Wilcoxon-rank sum test. ^*^p-value < 0.001; ^***^p-value < 10^−5^.

**Figure 4:**
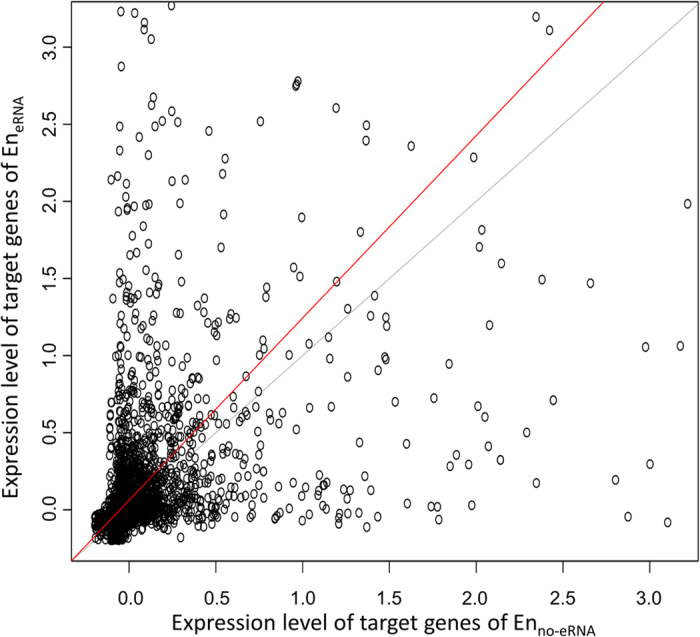
Expression level of the same target genes of the same positioned enhancers across tissues. *Z*-scores of the BPKM were calculated for each tissue. Each dot represents the average expressions (*z*-scores) of the same gene targeted by the same positioned En_eRNA_ or En_no-eRNA_ (see Material and Methods). The red line is least rectangles regression. The grey line is a reference diagonal line with a slope of one. For presentation, the axes are limited to three times the standard deviation of the average *z*-scores.

Based on the tight link between eRNAs and target gene expression, and the previous cases on eRNA as a positive contributor^9^,^10^,^11^,^12^,^13^, we then conducted a genome-wide examination on whether the presence of eRNA is associated with a higher expression level of the corresponding target genes. We compared the expression level of genes regulated by En_eRNA_ and those by En_no-eRNA_. The genes regulated by En_eRNA_ exhibit significantly higher expression level as compared to genes regulated by En_no-eRNA_ in all examined tissues (Fig. 3). To further confirm this difference in expression of target genes is due to the presence of eRNA, we ensured the same enhancers of different tissues also target the same genes, leaving the presence or absence of eRNAs as the only variation in the comparison. In other words, we specifically selected for groups of same positioned En_eRNA_ and En_no-eRNA_ across tissues targeting the same genes. These same positioned enhancers were grouped if they 1) reside within 1000 bp, 2) target the same genes, and 3) contain both En_eRNA_ and En_no-eRNA_ (see Methods). We then compared the expression level of the genes from these groups. The expression level of target genes of the En_eRNA_ tends to be higher than that of the En_no-eRNA_ and results of a paired two sample *t*-test also show that the expression of target genes of En_eRNA_ is significantly higher than that of the En_no-eRNA_ (*p*-value < 0.001, Fig. 4). We thus showed that even for the same enhancers and target genes, the presence of eRNA corresponds with a higher expression level of target genes. Therefore, the two states of enhancers, as distinguished by eRNAs, are indeed different in the expression of their target genes. This observation is not limited to specific cases, but is a genome-wide and cross-tissue phenomenon. Additionally, the existence of same positioned enhancers containing both En_eRNA_ and En_no-eRNA_ indicates that the same enhancers may be transcribing eRNAs in some tissues but not transcribing eRNAs in other tissues. Enhancers and eRNAs are known to be tissue-specific^2^,^27^,^28^, and there is a general dependency between tissue-specific enhancers and expression of eRNAs^28^. Our results reinforce this tissue specific dependency and further imply that transcription at enhancers may be controlled in a tissue-specific manner. Together, we show two states of enhancers exist as distinguished by the presence of eRNAs indicate a higher expression level of target genes on a global scale, and this presence is tissue-specific.

### Presence of eRNA indicates a higher number of tissue-specific function in the target genes of corresponding enhancer

The difference in gene expression based on presence of eRNA entailed an investigation on whether these genes also differs functionally. We conducted functional enrichment analysis by the DAVID tool^29^,^30^ on the target genes of En_eRNA_ and En_no-eRNA_ for each tissue. DAVID clusters similar Gene Ontology terms that share global gene profiles^29^,^30^. Only clusters with score ≥ 3 (corresponds to a negative log transformed *p*-value of <0.001) were selected. Considering only the unique annotations between the target genes of En_eRNA_ and En_no-eRNA_ (Table S2 and Supplementary Data), we discovered that target genes of En_eRNA_ are annotated with numerous clusters containing functional annotations terms directly related to its tissue. For example, BrainE14.5 is annotated with a cluster containing “brain development”-related terms, Heart with a cluster containing “cardiac muscle tissue morphogenesis”-related terms, Liver with a cluster containing “xenobiotic process”-related terms, and Spleen with a cluster containing “immune cell proliferation”-related terms. Conversely, target genes of En_no-eRNA_ are annotated with only a few clusters containing terms directly related to the tissue (Table S2 and Supplementary Data). The functional enrichment analysis reveals that target genes of En_eRNA_ are annotated with a higher number of tissue-specific functions. Previous studies have shown eRNAs to be tissue specific^27^,^28^, and possibly act as a positive contributor to gene transcription^9^,^10^,^11^,^12^,^13^. We further provided support that the genes targeted by En_eRNA_ are globally associated with a significantly higher expression and more tissue-specific functions, and eRNA production might be controlled in a tissue-specific manner. Based on previous results and our findings that the presence of eRNA indicate a higher level of expression in enhancer target genes, we provide a conjecture that eRNAs may be involved in the selective activation of genes in different tissues.

### Enhancer RNAs contain particular regions similar to miRNA in sequence and secondary structure

**Figure 5:**
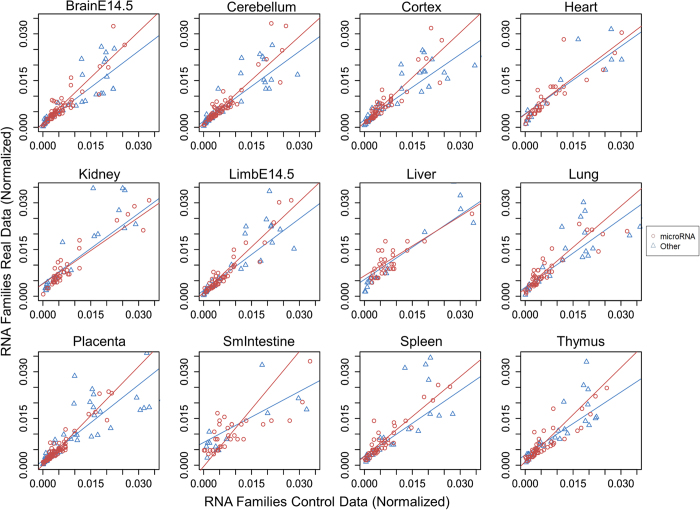
eRNA matches to miRNAs and other RNAs. Dots in each plot represent the significant test statistics of eRNAs matching to miRNAs (denoted by red circles) or other RNAs (denoted by blue triangles). Significances were estimated using a two-proportion *t*-test, which tests the eRNA matches to miRNA versus other RNAs against random intergenic control data. The trend lines are least rectangles regression for miRNA data (red) and other RNA data (blue). For presentation, all axes are limited to 0.35 and represent the ratio of hits to a specific RNA family to the total number of significant hits from Rfam.

Thus far, the target gene expression differences indicated by the presence of eRNAs has been confirmed, we next examine if these eRNAs have any similarity with known ncRNAs, which may provide clues to how eRNA might partake in regulation. We scanned our eRNAs with Infernal 1.1 of the Rfam database^31^. Infernal matches query sequences to known ncRNAs by a covariance model considering sequence, secondary structure, and conservation, with a particular focus in secondary structure. The scan resulted in the matching of many eRNAs to miRNAs families. Surprisingly, these eRNAs with regions similar to miRNAs account for over half of all family annotations from Infernal for each tissue. Henceforth, these eRNAs are denoted as miR-like eRNAs. Furthermore, the number of annotation matches is more significant when compared to annotation matches of random intergenic sequences by a two-sample proportion test (*p*-value < 0.001). Among the significant annotated families, the eRNA matches to miRNAs hold a higher proportion than the matches to other known RNA families as shown by the trend lines for 9 out of the 12 tissues considered (Fig. 5). For Heart, Kidney, and Liver, the trend lines are too close to make a definite determination. To make a statistical determination between the trend lines, we used another two-proportion *z*-test, where the matches to miRNAs versus random controls were compared to other non-coding RNA matches versus random controls. For the same 9 out of the 12 tissues the miRNAs the proportion of matches to miRNAs was higher (*p*-value < 0.001, Table S3). Interestingly, a recent experimental study which reports various known miRNAs can be transcribed from a super-enhancer region^32^ indeed supports our finding.

There is increasing evidence that a subset of miRNAs may also activate gene expression through targeting promoters^20^,^21^,^22^,^23^,^24^,^25^, although miRNA was previously thought to solely be a repressor. Therefore, these miR-like eRNAs warranted further investigation. We ensured the miR-like eRNAs contain their respective miRNA sequences with BLASTN. Then we scanned for target sites of these miR-like sequences in the promoters of their corresponding target genes by using TargetScan^33^ and miRanda^34^ separately. Since currently the eRNAs with known function are positive regulators, the targets were ensured to be unique to the promoters of the target genes and not the 3′ untranslated region more commonly associated the repressing ability of miRNA. This resulted in 69 matches to promoter regions, resulting in a total of 27 unique miRNA families (Table S4). Shuffling and bootstrapping of the each of the resulting miR-like-eRNAs 100 times in Cortex shows our results are not due to chance (both TargetScan and miRanda have a bootstrapping *p*-value < 0.001). We additionally verified the existence of a subset of the miR-like sequences in another RNA-seq dataset (ENCODE LICR) with shorter reads (35 bp) (Table S4). Note that we did not consider any reads that can be assembled into contigs longer than 76 bp, which is the length of the RNA-seq reads used to determine our eRNAs. We therefore showed that subset of the sequences matched with miRNAs in the miR-like-eRNAs exist in shorter RNA-seq data sets.

Our results show that some eRNAs have regions that are highly similar to miRNAs in sequence, secondary structure, and conservation. It is already known from Lam *et al.* that the sequences of eRNAs is important for its function and that modifying its sequence can affect its function^9^. However, it has also been found that only specific portions of the eRNA sequence are important for the function and other factors in addition to sequence may play a role in the functional viability of eRNAs^13^. Using Infernal considers other factors besides sequence, such as secondary structure and sequence position conservation, which supports similarity between the found eRNAs and known miRNA families. However, it is to be noted that a typical Rfam scan, which combines BLAST and Infernal simultaneously, is unsuitable for our study due to eRNAs being an entire novel set of ncRNAs. We nevertheless followed up Infernal by aligning the specific miR-like regions in the eRNAs with their respective miRNAs using BLASTN, showing that our miR-like eRNAs contain elements matching to miRNA seeds and miRNA mature sequences. Adding that these elements exist in RNA-seq datasets with shorter reads, we provide numerous supports for the existence of miR-like eRNAs. Moreover, while TargetScan and miRanda are the two most widely used miRNA target discovery tools, they are typically used for detecting miRNAs target sites in 3′ UTRs. For the purpose of our study, where we require the complementarity of eRNAs to promoters, TargetScan and miRanda were chosen for the found similarity between eRNAs and miRNAs and as a method for more sophisticated prediction than simple sequence complementation.

The possibility that eRNAs may have a positive contribution to gene expression have been widely speculated. Earlier research has found that a set of ncRNAs have enhancer-like properties and up-regulate genes^17^. Subsequent studies showed that eRNAs are important with regards to the looping mechanism necessary for enhancer and promoter interaction^7^,^9^, and that eRNAs may play a role in the recruitment of RNA Polymerase II^9^. All of these finding suggest that eRNAs are transcribed before transcription of genes^13^,^35^, thus raising the possibility of eRNAs contributing to the later transcription process. More confirmatory results in multiple tissues of human and mouse show knocking down eRNAs also reduces target gene expression^9^,^10^,^11^,^12^,^13^. Thus, combining the findings from previous studies and our results further provides evidence for the supposition that these miR-like eRNAs may have gene activating potential in the cell.

## Conclusion

This study investigates the role of eRNA in enhancer-regulated transcription in a genome-wide scale across 12 mouse tissues. Our results show that enhancers transcribing and not-transcribing eRNAs (i.e. En_eRNA_ and En_no-eRNA_) exist in proportionate numbers, indicating the transcription of eRNAs might separate enhancers into two states. The two states of enhancers may regulate different gene sets with different expression and function. The expression level of target genes of En_eRNA_ is higher as compared to that of En_no-eRNA_, in accordance with previous findings that eRNAs positively and contributes to enhancer-regulated transcription^9^,^10^,^11^,^12^,^13^. Moreover, the function of the target genes of En_eRNA_ tends to be more enriched with processes specific to the examined tissue. Further supporting the eRNAs may play a role in tissue-specific control. These effects of eRNAs on expression and tissue-specific control are similar to other ncRNAs with activating potential. We indeed found that a large number of eRNAs contain particular regions similar to miRNAs, which have been discovered to sometimes act as an activator rather than a repressor when targeting promoters^20^,^21^. Interestingly, some target promoters of the enhancers are found to contain complementary regions to these the miRNA-like regions in the respective eRNAs. While experimental confirmation of the proposed idea is required, we nevertheless showed the existence of two states of enhancers as distinguished by eRNAs, and demonstrate that eRNAs indicate the existence of an additional layer of positive regulation to control the expression of the target genes.

## Methods

### Enhancer retrieval and eRNA identification

Genomic coordinates of enhancers and target genes for 12 mouse tissues were obtained from Shen *et al.*^26^. Shen *et al.* identified enhancers and promoters by correlating the ChIP-seq signals of histone markers, coactivator (at enhancer), and RNA polymerase II (at promoter). The enhancers and promoters are then paired by correlating the ChIP-seq signals if they both reside within a defined genome block, which is based on ChIP-seq signals of CTCF, histone markers and RNA polymerase II. A subset of the enhancer and promoters were verified by luciferase assay, 3C and Hi-C experiments, and matching with Ref-Seq annotated promoters. To avoid confusion between transcripts from enhancers and transcripts of any known gene, we only examined intergenic enhancers that are 3 k bp away from any start or end of genes recorded in the RefSeq Gene database^36^. The RNA-seq data of the 12 examined tissues were retrieved from the ENCODE CSHL Long RNA-seq datasets^37^. For each tissue, ENCODE provides a set of RNA contigs, which were assembled from continuous regions covered by uniquely aligned reads allowing 25 bp of gaps from merging two replicates of RNA-seq experiments for each tissue. To identify enhancers that transcribe eRNAs, we modified the eRNA identification method by ENCODE^14^. We considered an RNA contig as an eRNA if its transcript start (*i.e.* 5′ end) fell within a 3 k bp upstream and downstream regions around the enhancer locus. To further avoid confusion between the eRNAs that arise from known genes or from intergenic regions, we removed the eRNAs that overlap with any known genes.

### Expression correlation of eRNA and the corresponding enhancer’s target gene

The expression correlation between an eRNA and the target gene of the corresponding enhancer is calculated by Pearson correlation. The expression level of the target gene was determined as the BPKM (bases per kilobase of gene model per million mapped bases^38^) of the region starting from the gene start site and spanning the same length as its paired eRNA. The expression level of the eRNA was also defined by BPKM. To examine the possible biases due to regions highly transcribed RNA polymerase II, we subsequently compared the expression level to that of the enhancer flanking regions. The enhancer flanking regions are regions starting from the upstream (resp. downstream) boundary of the 3 k region around the enhancer locus and extending for 0–1 k, 1–2 k, and 2–3 k. If the end of the eRNA extends beyond the 3 k region around the enhancer, the end of the eRNA was used as the starting boundary. Any case where the enhancer flanking region overlapped with a known gene was not considered for comparison.

### Expression difference of the target genes based on the presence of eRNA

The expression level between the target genes associated with transcribing and non-transcribing enhancers were compared. Enhancers of the 12 tissues targeting the same gene were selected, and those residing within a 1000 bp window were grouped. Each group needed to contain at least one transcribing and one non-transcribing enhancers. For different groups that target the same gene, the group with maximum number of enhancers was selected for expression analysis. If more than one group has the maximum number of enhancers, one group was randomly selected among the groups containing the same enhancer; the groups containing entirely different enhancers were considered as separate groups. Thus, each group contains transcribing and non-transcribing enhancers that are from different tissues and target the same gene. The expressions of target genes of transcribing enhancers were then compared to those of non-transcribing enhancers in each group. The expression levels were standardized as the *z*-scores of the BPKMs. Note that the expressions (*z*-scores) of target genes of transcribing enhancers (or non-transcribing enhancers) from different tissues in the same group were averaged.

### Annotations of eRNAs using known ncRNA families

The web-tool Infernal 1.1 of the Rfam database^39^ was used to match eRNAs with known ncRNA families in *Mus musculus*. Infernal matches query sequence to known ncRNAs by a covariance model that considers sequence, secondary structure and conservation. Our matches were with settings only allowing for global matches and non-truncated matches to improve the reliability of the results. To filter for significant results, the same number of random sequences as the number of eRNAs was chosen from intergenic regions, each with the same length as a randomly chosen eRNA and were run through Infernal. Using a one-sided two-sample proportion test, the proportions of Infernal annotated eRNAs were tested against the proportion of annotated random sequences for each ncRNA family. Results with *p*-values < 0.001 were selected as significant annotations.

### Identification of miRNA-like target site of eRNA

We modified the method by Place *et al.*^21^ to examine if eRNA has potential target sites in the promoters of the corresponding enhancer target genes. The miR-Family data from TargetScan^33^ was used to obtain mature miRNA sequences and miRNA seeds of *Mus musculus*. We selected the eRNAs that contain regions similar to miRNA families as determined by Infernal, and designated them as miRNA-like eRNAs. Using BLASTN, the miRNA-like eRNAs were matched with miRNA seed from TargetScan or mature miRNA sequences from miRBase, and those that has 100% identity with the same annotated miRNA from Infernal were kept. Using TargetScan and miRanda, each miRNA seed and mature miRNA, respectively, was scanned against the sense and antisense promoter sequences of potential target genes of the eRNA to find potential target sites. The promoter region was defined as ≤1000 bp upstream to 200 bp downstream intergenic region of a gene start, with any segment that overlapped with the gene body of another gene removed. Only eRNAs with potential target sites in the promoter region of target genes but not in the 3′ untranslated region (3′ UTR; defined as from the end of the coding region to the gene end) of the gene were considered in our analysis. Additionally, the miR-like region in the eRNAs was matched with another RNA-seq dataset with shorter reads (35 bp), the ENCODE LICR dataset, to verify their existence. Only the reads that are assembled to be shorter than 76 bp, which is the length of ENCODE CHSL long RNA-seq reads, was considered. As a control, we shuffled the sequences of the resulting miR-like eRNA and bootstrapped for 100 times each. Then the exact same identification method of miRNA-like target site was conducted with the shuffled sequences, and the bootstrapping *p*-value was calculated.

## Additional Information

**How to cite this article**: Cheng, J.-H. *et al.* Genome-wide analysis of enhancer RNA in gene regulation across 12 mouse tissues. *Sci. Rep.* **5**, 12648; doi: 10.1038/srep12648 (2015).

## Supplementary Material

Supplementary Information
