# Supplementary material for: Genome-wide analysis of enhancer RNA in gene regulation across 12 mouse tissues
Source: Sci Rep. 2015 Jul 29;5:12648. doi: 10.1038/srep12648 (PMC4518263; doi:10.1038/srep12648)
Supplement: Supplementary Information [file srep12648-s1.pdf]

Supplementary Information

Genome-wide analysis of enhancer RNA in gene regulation across 12 mouse tissues

Jen-Hao Cheng, David Zhi-Chao Pan, Zing Tsung-Yeh Tsai, and Huai-Kuang Tsai*

Institute of Information Science, Academia Sinica, 128 Academia Road, Section 2, Nankang, Taipei 115, Taiwan

*To whom correspondence may be addressed:

Huai-Kuang Tsai

Institute of Information Science, Academia Sinica
128 Academia Road, Section 2
Nankang, Taipei 115
Taiwan
(T):886-2-27883799 ext 1718
(F): 886-2-27824814
Email: hktsai@iis.sinica.edu.tw

This file includes:

Supplementary Tables S1 to S4

Supplementary Table S1. No correlation between expression levels of enhancers and control regions. The gene upstream regions are the intergenic region upstream of the gene start site with the same length as its paired eRNA. The random intergenic regions are intergenic regions that do not overlap with the 3k region around the enhancer locus, and they are of the same length and chromosome as the paired eRNA.

Tissue	Pearson correlation (ρ) of enhancer and gene upstream region	*p*-value	Pearson correlation (ρ) of enhancer and random intergenic region	*p*-value
BrainE14.5	-3.4×10^{-3}	-0.59	-1.5×10^{-3}	-0.82
Cerebellum	-4.8×10^{-3}	-0.55	-9.2×10^{-4}	-0.91
Cortex	-3.4×10^{-3}	-0.62	-1.2×10^{-3}	-0.86
Heart	1.3×10^{-3}	-0.89	2.6×10^{-4}	-0.98
Kidney	-2.7×10^{-3}	-0.70	-1.1×10^{-3}	-0.88
LimbE14.5	-9.1×10^{-4}	-0.89	-1.0×10^{-3}	-0.87
Liver	-4.2×10^{-3}	-0.68	-1.6×10^{-3}	-0.88
Lung	-3.1×10^{-3}	-0.64	-9.6×10^{-4}	-0.88
Placenta	-8.4×10^{-4}	-0.84	-4.6×10^{-4}	-0.91
SmIntestine	-2.3×10^{-3}	-0.79	-2.4×10^{-3}	-0.78
Spleen	-2.8×10^{-3}	-0.68	-1.8×10^{-3}	-0.79
Thymus	-2.0×10^{-3}	-0.74	3.4×10^{-3}	-0.57

Supplementary Table S2. Unique functional annotation terms between target genes of En_{eRNA} and $En_{no\text{-}eRNA}$. Functional enrichment was determined by DAVID Functional Annotation Cluster, which groups similar GO terms into one cluster. Only clusters with scores ≥ 3 (correspond to the negative log of *p*-value ≤ 0.001) were considered. Same GO terms between target genes of En_{eRNA} and $En_{no\text{-}eRNA}$ of the same tissues were removed, leaving only terms unique to each enhancer state (see Supplementary Data). For a simpler presentation, we constructed a summary table. The summary table presents generalized annotation terms for each cluster that retained more than half of the original GO terms before removal. Bold text with gray background is terms directly related to the tissue; black text is terms of non-general function; gray text is terms of general function.

Tissue	Target Genes of En_{eRNA}	Score	Target Genes of $En_{no\text{-}eRNA}$	Score
BrainE14.5	Phosphorylation	4.29	Negative regulation of gene-specific transcription	3.78
	Cell death	3.92		
	Limb development	3.88		
	Positive regulation of cell motion	3.43		
	Brain development	3.30		
	Kidney development	3.17		
	Ventral spinal cord development	3.08		
Cerebellum	Catabolic processes	4.90	Neuron development	4.55
	Phosphorylation	4.04	Positive regulation of cell development	3.58
	Cell death	3.86		
Cortex	Ion transport	3.97		
	Endocytosis	3.28		
Heart	Cardiac muscle tissue morphogenesis	5.96	Epithelial tube development	5.68
			Phosphorylation	5.46
			Positive regulation of cell motion	5.10
			Embryonic development	4.73
			Actin filament-based process	4.08
			Regulation of cell death	3.37
			Positive regulation of cell adhesion	3.10
Kidney			Regulation of cell death	5.13
			Pattern specification	4.38
			Mammary gland development	3.71
LimbE14.5	Pattern specification	9.40	Salivary gland development	4.76
	Bone development	6.82	Eye development	3.15
	Cell death	5.71		
	Mesoderm development	4.05		

	Prostate gland development	3.81		
	Macromolecule catabolic processes	3.60		
	Reproductive developmental processes	3.56		
	Regulation of cell motion	3.41		
	Regulation of phospho-group metabolic processes	3.40		
	Neural tube development	3.15		
	Chromatin assembly	3.08		
	Regulation of cell death	3.08		
	mRNA metabolic processes	3.06		
	Cell adhesion	3.04		
Liver	Immune response	4.05	Vasculature development	9.04
	Xenobiotic related processes	3.18	Regulation of cell death	4.88
	Response to nutrients	3.15	Cell death	4.59
			Epithelial tube development	4.52
			Gland development	4.34
			Phosphorylation	4.00
			Lung development	3.64
			Negative regulation of transcription	3.61
			Coenzyme and cofactor metabolic process	3.61
			Sugar metabolic processes	3.05
Lung			Positive regulation of signal transduction	6.35
			Negative regulation of kinase activity	5.09
			Embryonic development	4.80
			Positive regulation of cytokine production	4.65
			Positive regulation of kinase activity	4.61
			Cell motion	4.31
			Inflammatory response	4.07
			Positive regulation of cell adhesion	3.56
			Positive regulation of myeloid cell differentiation	3.43
			Immune-response signaling pathway	3.40
			Positive regulation of protein kinase cascade	3.16
			Actin filament-based process	3.06

Placenta*	Negative regulation of transcription	6.52	Embryonic development	4.97
	Cell death	4.26	Epithelial Tube development	3.41
	Phosphorylation	4.21		
	Protein catabolic process	3.486		
SmIntestine*	Epithelial tube development	3.78	Regulation of myeloid cell differentiation	4.50
	Placenta development	3.48	Sugar metabolic process	3.39
	Response to sugar stimulus	3.27	Cell motion	3.08
Spleen	Positive regulation of organelle organization	3.26	Regulation of myeloid cell differentiation	5.42
	Immune cell proliferation	3.16	Positive regulation of interleukin-2 production	5.21
	Antigen processing and presentation	3.03	Chromatin assembly	4.69
			Lung development	3.70
			Positive regulation of protein metabolic processes	3.47
			Erythrocyte homeostasis	3.22
Thymus	Positive regulation of protein kinase cascade	4.42	Negative regulation of transcription	6.49
	Immune cell selection	4.14	Embryonic development	3.96
	Immune-response related signaling pathway	3.42	Pattern specification	3.84
	Erythrocyte homeostasis	3.06	Receptor protein signaling pathway	3.69
			Vasculature development	3.54

* En_{eRNA} in Placenta and $En_{no\text{-}eRNA}$ for SmIntestine target more than 3000 genes, which could not be input into DAVID. We randomly selected 3000 genes for 10 times, and determined the functional annotation clusters for each repetition. Only the cluster with an average score ≥ 3 over the 10 repetitions were considered. The GO terms within each cluster needs to be enriched in all 10 repetitions.

Unique functional annotation terms between En-eRNA and En-no-eRNA in BrainE14.5

En-eRNA	Score	En-no-eRNA	Score
**Annotation Cluster**	7.139310564	**Annotation Cluster**	3.783843
neuron migration		regulation of specific transcription from RNA polymerase II promoter	
**Annotation Cluster**	5.031927107	negative regulation of specific transcription from RNA polymerase II promoter	
epithelial tube morphogenesis		negative regulation of gene-specific transcription	
**Annotation Cluster**	4.327702526	regulation of gene-specific transcription	
embryonic cranial skeleton morphogenesis		**Annotation Cluster**	3.571625
**Annotation Cluster**	4.28918518	negative regulation of neurogenesis	
phosphorus metabolic process		negative regulation of cell development	
phosphate metabolic process		negative regulation of neuron differentiation	
protein amino acid phosphorylation			
phosphorylation			
**Annotation Cluster**	3.922473753		
apoptosis			
programmed cell death			
death			
cell death			
**Annotation Cluster**	3.884688379		
embryonic appendage morphogenesis			
embryonic limb morphogenesis			
limb development			
appendage development			
appendage morphogenesis			
limb morphogenesis			
hindlimb morphogenesis			
**Annotation Cluster**	3.425614939		
regulation of cell motion			
regulation of cell migration			
regulation of locomotion			
positive regulation of cell motion			
positive regulation of cell migration			
positive regulation of locomotion			
**Annotation Cluster**	3.296699989		
telencephalon development			
limbic system development			
pallium development			
hippocampus development			
cerebral cortex development			
**Annotation Cluster**	3.170556112		
urogenital system development			
kidney development			
ureteric bud development			
metanephros development			
**Annotation Cluster**	3.084253687		
cell fate commitment			
neuron fate commitment			
cell fate specification			
neuron fate specification			
spinal cord development			
dorsal spinal cord development			
spinal cord association neuron differentiation			
spinal cord patterning			
cell differentiation in spinal cord			
ventral spinal cord interneuron fate commitment			
ventral spinal cord interneuron specification			
ventral spinal cord interneuron differentiation			
spinal cord dorsal/ventral patterning			
spinal cord motor neuron cell fate specification			
ventral spinal cord development			
spinal cord motor neuron differentiation			

Unique functional annotation terms between En-eRNA and En-no-eRNA in Cerebellum

En-eRNA	Score	En-no-eRNA	Score
**Annotation Cluster**	4.906236	**Annotation Cluster**	4.553524
protein catabolic process		cell projection organization	
proteolysis involved in cellular protein catabolic process		neuron projection morphogenesis	
cellular macromolecule catabolic process		neuron projection development	
cellular protein catabolic process		cell morphogenesis	
macromolecule catabolic process		cell morphogenesis involved in neuron differentiation	
modification-dependent macromolecule catabolic process		neuron differentiation	
modification-dependent protein catabolic process		cell morphogenesis involved in differentiation	
proteolysis		neuron development	
**Annotation Cluster**	4.038438	cell projection morphogenesis	
phosphate metabolic process		cell part morphogenesis	
phosphorus metabolic process		axonogenesis	
protein amino acid phosphorylation		cellular component morphogenesis	
phosphorylation		cell motion	
**Annotation Cluster**	3.858178	axon guidance	
programmed cell death		**Annotation Cluster**	3.582627
apoptosis		regulation of cell development	
death		positive regulation of developmental process	
cell death		positive regulation of cell differentiation	

Unique functional annotation terms between En-eRNA and En-no-eRNA in Cortex

En-eRNA	Score	En-no-eRNA	Score
**Annotation Cluster**	3.968857	NA	
potassium ion transport			
ion transport			
metal ion transport			
cation transport			
monovalent inorganic cation transport			
calcium ion transport			
di-, tri-valent inorganic cation transport			
**Annotation Cluster**	3.275092		
membrane organization			
vesicle-mediated transport			
endocytosis			
membrane invagination			

Unique functional annotation terms between En-eRNA and En-no-eRNA in Heart

En-eRNA	Score	En-no-eRNA	Score
**Annotation Cluster**	5.957664	**Annotation Cluster**	5.679096
heart development		morphogenesis of a branching structure	
heart morphogenesis		branching morphogenesis of a tube	
muscle tissue morphogenesis		tube morphogenesis	
cardiac muscle tissue morphogenesis		epithelium development	
ventricular septum morphogenesis		gland development	
heart septum morphogenesis		tissue morphogenesis	
		morphogenesis of an epithelium	
		epithelial tube morphogenesis	
		**Annotation Cluster**	5.46426
		phosphorus metabolic process	
		phosphate metabolic process	
		phosphorylation	
		protein amino acid phosphorylation	
		**Annotation Cluster**	5.099076
		regulation of cell motion	
		regulation of cell migration	
		regulation of locomotion	
		positive regulation of cell motion	
		positive regulation of cell migration	
		positive regulation of locomotion	
		**Annotation Cluster**	4.725256
		in utero embryonic development	
		chordate embryonic development	
		embryonic development ending in birth or egg hatching	
		embryonic organ development	
		embryonic morphogenesis	
		embryonic organ morphogenesis	
		**Annotation Cluster**	4.076574
		actin cytoskeleton organization	
		actin filament-based process	
		actin filament organization	
		cytoskeleton organization	
		**Annotation Cluster**	3.749788
		muscle cell development	
		skeletal muscle tissue development	
		skeletal muscle organ development	
		**Annotation Cluster**	3.371172
		regulation of programmed cell death	
		regulation of cell death	
		regulation of apoptosis	
		positive regulation of programmed cell death	
		positive regulation of cell death	
		positive regulation of apoptosis	
		negative regulation of programmed cell death	
		negative regulation of cell death	
		negative regulation of apoptosis	
		induction of programmed cell death	
		induction of apoptosis	
		anti-apoptosis	
		**Annotation Cluster**	3.099431
		regulation of cell adhesion	
		positive regulation of cell adhesion	
		regulation of cell-substrate adhesion	
		positive regulation of cell-substrate adhesion	

Unique functional annotation terms between En-eRNA and En-no-eRNA in Kidney

En-eRNA	Score	En-no-eRNA	Score
NA		**Annotation Cluster**	5.132835
		regulation of programmed cell death	
		regulation of cell death	
		regulation of apoptosis	
		positive regulation of apoptosis	
		positive regulation of programmed cell death	
		positive regulation of cell death	
		negative regulation of programmed cell death	
		negative regulation of cell death	
		negative regulation of apoptosis	
		induction of programmed cell death	
		induction of apoptosis	
		anti-apoptosis	
		**Annotation Cluster**	4.383481
		embryonic skeletal system development	
		embryonic skeletal system morphogenesis	
		anterior/posterior pattern formation	
		embryonic organ morphogenesis	
		skeletal system development	
		pattern specification process	
		skeletal system morphogenesis	
		regionalization	
		**Annotation Cluster**	3.708397
		gland development	
		mammary gland development	
		gland morphogenesis	
		mammary gland morphogenesis	
		branching involved in mammary gland duct morphogenesis	
		mammary gland duct morphogenesis	

Unique functional annotation terms between En-eRNA and En-no-eRNA in LimbE14.5

En-eRNA	Score	En-no-eRNA	Score
**Annotation Cluster**	9.402845	**Annotation Cluster**	4.763031
pattern specification process		exocrine system development	
regionalization		salivary gland morphogenesis	
anterior/posterior pattern formation		salivary gland development	
**Annotation Cluster**	7.132038	branching involved in salivary gland morphogenesis	
mammary gland duct morphogenesis		**Annotation Cluster**	4.463635
branching involved in mammary gland duct morphogenesis		embryonic digit morphogenesis	
**Annotation Cluster**	6.817372	**Annotation Cluster**	4.200049
skeletal system development		regulation of morphogenesis of a branching structure	
bone development		developmental growth involved in morphogenesis	
ossification		branch elongation of an epithelium	
osteoblast differentiation		**Annotation Cluster**	3.847595
**Annotation Cluster**	5.706563	odontogenesis	
apoptosis		odontogenesis of dentine-containing tooth	
programmed cell death		**Annotation Cluster**	3.146912
cell death		eye morphogenesis	
death		camera-type eye morphogenesis	
**Annotation Cluster**	4.054998	camera-type eye development	
gastrulation		eye development	
mesoderm development		**Annotation Cluster**	3.010128
mesoderm morphogenesis		regulation of MAPKKK cascade	
formation of primary germ layer		positive regulation of MAPKKK cascade	
mesoderm formation		regulation of protein kinase cascade	
**Annotation Cluster**	3.812448		
reproductive structure development			
**Annotation Cluster**	3.601106		
macromolecule catabolic process			
cellular macromolecule catabolic process			
protein catabolic process			
modification-dependent protein catabolic process			
modification-dependent macromolecule catabolic process			
proteolysis involved in cellular protein catabolic process			
cellular protein catabolic process			
proteolysis			
**Annotation Cluster**	3.55816		
reproductive developmental process			
reproductive structure development			
sex differentiation			
**Annotation Cluster**	3.405734		
regulation of cell motion			
regulation of cell migration			
regulation of locomotion			
positive regulation of cell motion			
positive regulation of cell migration			
positive regulation of locomotion			
**Annotation Cluster**	3.396329		
regulation of phosphate metabolic process			
regulation of phosphorus metabolic process			
regulation of cellular protein metabolic process			
regulation of protein amino acid phosphorylation			
regulation of protein modification process			
**Annotation Cluster**	3.146775		
neural tube development			
morphogenesis of embryonic epithelium			
tube lumen formation			
neural tube formation			
primary neural tube formation			
embryonic epithelial tube formation			
neural tube closure			
tube closure			
**Annotation Cluster**	3.082192		
cellular macromolecular complex subunit organization			
nucleosome organization			
nucleosome assembly			
cellular macromolecular complex assembly			
chromatin assembly			
protein-DNA complex assembly			
chromatin assembly or disassembly			
DNA packaging			
**Annotation Cluster**	3.082192		
regulation of cell death			
regulation of programmed cell death			
regulation of apoptosis			
positive regulation of cell death			
positive regulation of apoptosis			
positive regulation of programmed cell death			
induction of apoptosis			
induction of programmed cell death			
negative regulation of programmed cell death			
negative regulation of cell death			
negative regulation of apoptosis			
anti-apoptosis			
**Annotation Cluster**	3.058044		
mRNA metabolic process			
mRNA processing			
RNA splicing			
RNA processing			
**Annotation Cluster**	3.039369		
cell adhesion			
biological adhesion			
cell-cell adhesion			

Unique functional annotation terms between En-eRNA and En-no-eRNA in Liver

**En-eRNA**	**Score**
**Annotation Cluster**	4.057788
acute inflammatory response	
activation of plasma proteins involved in acute inflammatory response	
complement activation	
humoral immune response mediated by circulating immunoglobulin	
complement activation, classical pathway	
humoral immune response	
immune effector process	
protein maturation by peptide bond cleavage	
activation of immune response	
leukocyte mediated immunity	
lymphocyte mediated immunity	
B cell mediated immunity	
protein maturation	
protein processing	
positive regulation of response to stimulus	
immunoglobulin mediated immune response	
positive regulation of immune system process	
adaptive immune response	
adaptive immune response based on somatic recombination of immune r	
positive regulation of immune response	
**Annotation Cluster**	3.181348
response to xenobiotic stimulus	
xenobiotic metabolic process	
benzene and derivative metabolic process	
**Annotation Cluster**	3.150501
response to extracellular stimulus	
response to nutrient levels	
response to nutrient	

**En-no-eRNA**	**Score**
**Annotation Cluster**	9.038732
vasculature development	
blood vessel development	
angiogenesis	
blood vessel morphogenesis	
**Annotation Cluster**	4.883672
regulation of apoptosis	
regulation of programmed cell death	
regulation of cell death	
positive regulation of programmed cell death	
positive regulation of cell death	
positive regulation of apoptosis	
negative regulation of apoptosis	
negative regulation of programmed cell death	
negative regulation of cell death	
anti-apoptosis	
induction of programmed cell death	
induction of apoptosis	
**Annotation Cluster**	4.590121
cell death	
death	
programmed cell death	
apoptosis	
**Annotation Cluster**	4.517877
tube development	
morphogenesis of a branching structure	
branching morphogenesis of a tube	
epithelium development	
tissue morphogenesis	
tube morphogenesis	
morphogenesis of an epithelium	
epithelial tube morphogenesis	
**Annotation Cluster**	4.336412
morphogenesis of a branching structure	
gland development	
gland morphogenesis	
**Annotation Cluster**	3.995015
phosphate metabolic process	
phosphorus metabolic process	
phosphorylation	
protein amino acid phosphorylation	
**Annotation Cluster**	3.635267
tube development	
lung alveolus development	
respiratory tube development	
lung development	
respiratory system development	
**Annotation Cluster**	3.611561
negative regulation of transcription, DNA-dependent	
negative regulation of RNA metabolic process	
negative regulation of transcription	
negative regulation of nucleobase, nucleoside, nucleotide and nucleic acid metabolic process	
negative regulation of transcription from RNA polymerase II promoter	
negative regulation of cellular biosynthetic process	
negative regulation of macromolecule biosynthetic process	
negative regulation of nitrogen compound metabolic process	
negative regulation of biosynthetic process	
negative regulation of gene expression	
negative regulation of macromolecule metabolic process	
**Annotation Cluster**	3.611396
coenzyme metabolic process	
cofactor metabolic process	
coenzyme biosynthetic process	
cofactor biosynthetic process	
**Annotation Cluster**	3.049875
monosaccharide metabolic process	
hexose metabolic process	
glucose metabolic process	

Unique functional annotation terms between En-eRNA and En-no-eRNA in Lung

En-eRNA	Score
**Annotation Cluster**	3.201494
regulation of B cell proliferation	
positive regulation of B cell proliferation	
regulation of CD4-positive, alpha beta T cell differentiation	
positive regulation of immune effector process	

En-no-eRNA	Score
**Annotation Cluster**	7.771605
gland development	
**Annotation Cluster**	6.415767
lung alveolus development	
**Annotation Cluster**	6.349062
positive regulation of signal transduction	
positive regulation of cell communication	
regulation of protein kinase cascade	
positive regulation of protein kinase cascade	
**Annotation Cluster**	5.089905
regulation of kinase activity	
regulation of transferase activity	
regulation of protein kinase activity	
negative regulation of molecular function	
negative regulation of protein kinase activity	
negative regulation of kinase activity	
negative regulation of transferase activity	
negative regulation of catalytic activity	
**Annotation Cluster**	4.805915
skeletal system development	
embryonic skeletal system morphogenesis	
skeletal system morphogenesis	
embryonic skeletal system development	
embryonic organ morphogenesis	
anterior/posterior pattern formation	
pattern specification process	
regionalization	
**Annotation Cluster**	4.648929
regulation of cytokine production	
positive regulation of cytokine biosynthetic process	
regulation of cytokine biosynthetic process	
**Annotation Cluster**	4.633614
myeloid cell differentiation	
**Annotation Cluster**	4.609679
positive regulation of molecular function	
regulation of kinase activity	
regulation of transferase activity	
regulation of protein kinase activity	
positive regulation of catalytic activity	
regulation of MAP kinase activity	
positive regulation of transferase activity	
positive regulation of kinase activity	
positive regulation of MAP kinase activity	
positive regulation of protein kinase activity	
activation of MAPK activity	
MAPKKK cascade	
activation of protein kinase activity	
**Annotation Cluster**	4.431853
cell migration	
cell motility	
localization of cell	
cell motion	
**Annotation Cluster**	4.073739
response to wounding	
inflammatory response	
defense response	
**Annotation Cluster**	3.555672
regulation of cell adhesion	
positive regulation of cell adhesion	
regulation of cell-substrate adhesion	
positive regulation of cell-substrate adhesion	
**Annotation Cluster**	3.524585
negative regulation of leukocyte activation	
negative regulation of cell activation	
negative regulation of lymphocyte activation	
negative regulation of immune system process	
negative regulation of T cell activation	
negative regulation of lymphocyte proliferation	
negative regulation of leukocyte proliferation	
negative regulation of mononuclear cell proliferation	
negative regulation of T cell proliferation	
negative regulation of B cell activation	
negative regulation of alpha-beta T cell proliferation	
negative regulation of B cell proliferation	
regulation of B cell differentiation	
**Annotation Cluster**	3.434174
regulation of myeloid cell differentiation	
positive regulation of myeloid cell differentiation	
positive regulation of myeloid leukocyte differentiation	
regulation of myeloid leukocyte differentiation	
**Annotation Cluster**	3.400385
positive regulation of response to stimulus	
immune response-regulating signal transduction	
immune response-activating signal transduction	
immune response-regulating cell surface receptor signaling pathway	
antigen receptor-mediated signaling pathway	
immune response-activating cell surface receptor signaling pathway	
positive regulation of immune response	
activation of immune response	
B cell receptor signaling pathway	
T cell receptor signaling pathway	
**Annotation Cluster**	3.157309
positive regulation of protein kinase cascade	
positive regulation of I-kappaB kinase/NF-kappaB cascade	
regulation of I-kappaB kinase/NF-kappaB cascade	
**Annotation Cluster**	3.055514
actin cytoskeleton organization	
actin filament-based process	
cytoskeleton organization	
actin filament organization	

Unique functional annotation terms between En-eRNA and En-no-eRNA in Placenta

**En-eRNA**	**Score**	**En-no-eRNA**	**Score**
**Annotation Cluster**	7.4586	**Annotation Cluster**	7.154782
positive regulation of nucleobase, nucleoside, nucleotide and nucleic acid meta		in utero embryonic development	
positive regulation of transcription, DNA-dependent		**Annotation Cluster**	7.048071
**Annotation Cluster**	6.516	regulation of transcription from RNA polymerase II promoter	
negative regulation of biosynthetic process		regulation of transcription	
negative regulation of cellular biosynthetic process		positive regulation of nucleobase, nucleoside, nucleotide and nucleic acid metabolic process	
negative regulation of nitrogen compound metabolic process		regulation of RNA metabolic process	
negative regulation of nucleobase, nucleoside, nucleotide and nucleic acid meta		regulation of transcription, DNA-dependent	
negative regulation of macromolecule biosynthetic process		positive regulation of transcription, DNA-dependent	
negative regulation of macromolecule metabolic process		transcription	
negative regulation of transcription		**Annotation Cluster**	4.971603
negative regulation of gene expression		skeletal system development	
negative regulation of transcription, DNA-dependent		embryonic skeletal system development	
negative regulation of RNA metabolic process		skeletal system morphogenesis	
negative regulation of transcription from RNA polymerase II promoter		embryonic skeletal system morphogenesis	
**Annotation Cluster**	4.264	pattern specification process	
apoptosis		embryonic organ morphogenesis	
programmed cell death		anterior/posterior pattern formation	
cell death		regionalization	
death		**Annotation Cluster**	3.412407
**Annotation Cluster**	4.207	epithelium development	
phosphate metabolic process		morphogenesis of a branching structure	
phosphorus metabolic process		tube morphogenesis	
protein amino acid phosphorylation		tissue morphogenesis	
phosphorylation		branching morphogenesis of a tube	
**Annotation Cluster**	3.58	morphogenesis of an epithelium	
placenta development		epithelial tube morphogenesis	
embryonic placenta development		**Annotation Cluster**	3.314985
labyrinthine layer development		lung alveolus development	
**Annotation Cluster**	3.486		
cellular macromolecule catabolic process			
macromolecule catabolic process			
proteolysis involved in cellular protein catabolic process			
cellular protein catabolic process			
modification-dependent protein catabolic process			
modification-dependent macromolecule catabolic process			
protein catabolic process			
proteolysis			

Unique functional annotation terms between En-eRNA and En-no-eRNA in SmIntestine

**En-eRNA**	**Score**	**En-no-eRNA**	**Score**
**Annotation Cluster**	7.039785	**Annotation Cluster**	5.777
regulation of transcription from RNA polymerase II promoter		negative regulation of nucleobase, nucleoside, nucleotide and nucleic acid metabolic process	
positive regulation of nucleobase, nucleoside, nucleotide and nucleic acid m		negative regulation of transcription, DNA-dependent	
positive regulation of transcription, DNA-dependent		**Annotation Cluster**	6.544
regulation of transcription		positive regulation of nucleobase, nucleoside, nucleotide and nucleic acid metabolic process	
transcription		positive regulation of transcription, DNA-dependent	
regulation of RNA metabolic process		**Annotation Cluster**	5.353
regulation of transcription, DNA-dependent		anti-apoptosis	
**Annotation Cluster**	5.038872	**Annotation Cluster**	4.498
embryonic organ development		regulation of myeloid cell differentiation	
embryonic morphogenesis		regulation of myeloid leukocyte differentiation	
**Annotation Cluster**	4.496149	**Annotation Cluster**	3.386
tube development		monosaccharide metabolic process	
**Annotation Cluster**	3.775234	hexose metabolic process	
tube development		glucose metabolic process	
tube morphogenesis		**Annotation Cluster**	3.077
epithelium development		cell migration	
epithelial tube morphogenesis		cell motion	
gland morphogenesis		localization of cell	
**Annotation Cluster**	3.5232	cell motility	
B cell differentiation			
**Annotation Cluster**	3.47739		
placenta development			
embryonic placenta development			
embryonic organ development			
labyrinthine layer development			
**Annotation Cluster**	3.26777		
response to carbohydrate stimulus			
response to glucose stimulus			
response to hexose stimulus			
response to monosaccharide stimulus			
**Annotation Cluster**	3.17325		
regulation of transcription from RNA polymerase II promoter			
negative regulation of nucleobase, nucleoside, nucleotide and nucleic acid metabolic process			
negative regulation of transcription, DNA-dependent			

Unique functional annotation terms between En-eRNA and En-no-eRNA in Spleen

**En-eRNA**	**Score**	**En-no-eRNA**	**Score**
**Annotation Cluster**	6.164712	**Annotation Cluster**	7.386221
regulation of adaptive immune response		anti-apoptosis	
regulation of adaptive immune response based on somatic recombination of immune receptors bui		**Annotation Cluster**	6.710421
positive regulation of cell differentiation		regulation of immune effector process	
regulation of B cell proliferation		regulation of B cell differentiation	
positive regulation of B cell proliferation		**Annotation Cluster**	5.420733
**Annotation Cluster**	3.26336	regulation of myeloid cell differentiation	
positive regulation of organelle organization		positive regulation of myeloid leukocyte differentiation	
regulation of organelle organization		regulation of myeloid leukocyte differentiation	
positive regulation of cellular component organization		positive regulation of myeloid cell differentiation	
**Annotation Cluster**	3.158063	**Annotation Cluster**	5.211148
mononuclear cell proliferation		positive regulation of interleukin-2 biosynthetic process	
leukocyte proliferation		regulation of interleukin-2 production	
lymphocyte proliferation		regulation of interleukin-2 biosynthetic process	
T cell proliferation		T cell differentiation in the thymus	
activated T cell proliferation		**Annotation Cluster**	4.848771
cell proliferation		negative regulation of cell activation	
**Annotation Cluster**	3.033479	negative regulation of leukocyte activation	
antigen processing and presentation of peptide antigen		negative regulation of immune system process	
antigen processing and presentation		negative regulation of lymphocyte activation	
antigen processing and presentation of peptide or polysaccharide antigen via MHC class II		negative regulation of T cell activation	
antigen processing and presentation of peptide antigen via MHC class II		negative regulation of T cell proliferation	
antigen processing and presentation of exogenous peptide antigen via MHC class II		negative regulation of lymphocyte proliferation	
antigen processing and presentation of exogenous peptide antigen		negative regulation of leukocyte proliferation	
chaperone mediated protein folding requiring cofactor		negative regulation of mononuclear cell proliferation	
antigen processing and presentation of exogenous antigen		negative regulation of B cell activation	
'de novo' protein folding		**Annotation Cluster**	4.685563
'de novo' posttranslational protein folding		nucleosome organization	
protein folding		nucleosome assembly	
		chromatin assembly	
		macromolecular complex subunit organization	
		protein-DNA complex assembly	
		macromolecular complex assembly	
		DNA packaging	
		chromatin assembly or disassembly	
		cellular macromolecular complex subunit organization	
		cellular macromolecular complex assembly	
		chromatin organization	
		chromosome organization	
		protein complex biogenesis	
		protein complex assembly	
		protein oligomerization	
		cellular protein complex assembly	
		**Annotation Cluster**	3.696091
		lung development	
		respiratory tube development	
		respiratory system development	
		tube development	
		lung alveolus development	
		**Annotation Cluster**	3.474047
		positive regulation of protein metabolic process	
		regulation of cellular protein metabolic process	
		regulation of peptidyl-tyrosine phosphorylation	
		positive regulation of cellular protein metabolic process	
		positive regulation of peptidyl-tyrosine phosphorylation	
		regulation of protein amino acid phosphorylation	
		regulation of protein modification process	
		positive regulation of protein modification process	
		positive regulation of phosphorylation	
		positive regulation of phosphate metabolic process	
		positive regulation of phosphorus metabolic process	
		positive regulation of protein amino acid phosphorylation	
		**Annotation Cluster**	3.221171
		myeloid cell differentiation	
		homeostasis of number of cells	
		erythrocyte homeostasis	
		erythrocyte differentiation	

Unique functional annotation terms between En-eRNA and En-no-eRNA in Thymus

**En-eRNA**	**Score**
**Annotation Cluster**	10.39695
B cell differentiation	
**Annotation Cluster**	5.128524
chromatin modification	
**Annotation Cluster**	4.415116
positive regulation of signal transduction	
positive regulation of cell communication	
regulation of protein kinase cascade	
positive regulation of protein kinase cascade	
**Annotation Cluster**	4.135392
thymic T cell selection	
T cell differentiation in the thymus	
T cell selection	
negative T cell selection	
negative thymic T cell selection	
positive T cell selection	
positive thymic T cell selection	
**Annotation Cluster**	3.703411
negative regulation of immune system process	
negative regulation of lymphocyte activation	
negative regulation of leukocyte activation	
negative regulation of cell activation	
negative regulation of T cell activation	
positive regulation of cell differentiation	
positive regulation of alpha-beta T cell proliferation	
negative regulation of T cell proliferation	
negative regulation of leukocyte proliferation	
negative regulation of lymphocyte proliferation	
negative regulation of mononuclear cell proliferation	
negative regulation of B cell activation	
**Annotation Cluster**	3.422271
immune response-regulating cell surface receptor signaling pathway	
antigen receptor-mediated signaling pathway	
immune response-regulating signal transduction	
regulation of antigen receptor-mediated signaling pathway	
immune response-activating cell surface receptor signaling pathway	
T cell receptor signaling pathway	
immune response-activating signal transduction	
positive regulation of response to stimulus	
positive regulation of immune response	
activation of immune response	
**Annotation Cluster**	3.062898
homeostasis of number of cells	
erythrocyte differentiation	
erythrocyte homeostasis	

**En-no-eRNA**	**Score**
**Annotation Cluster**	6.487863
negative regulation of transcription, DNA-dependent	
negative regulation of RNA metabolic process	
negative regulation of transcription from RNA polymerase II promoter	
negative regulation of nucleobase, nucleoside, nucleotide and nucleic acid metabolic process	
negative regulation of nitrogen compound metabolic process	
negative regulation of transcription	
negative regulation of cellular biosynthetic process	
negative regulation of macromolecule metabolic process	
negative regulation of gene expression	
negative regulation of biosynthetic process	
negative regulation of macromolecule biosynthetic process	
**Annotation Cluster**	3.957287
embryonic morphogenesis	
embryonic organ development	
embryonic development ending in birth or egg hatching	
chordate embryonic development	
in utero embryonic development	
**Annotation Cluster**	3.84285
embryonic morphogenesis	
pattern specification process	
anterior/posterior pattern formation	
regionalization	
**Annotation Cluster**	3.691139
transforming growth factor beta receptor signaling pathway	
enzyme linked receptor protein signaling pathway	
transmembrane receptor protein serine/threonine kinase signaling pathway	
**Annotation Cluster**	3.67559
protein complex biogenesis	
protein complex assembly	
protein oligomerization	
**Annotation Cluster**	3.537994
vasculature development	
blood vessel development	
blood vessel morphogenesis	
angiogenesis	
**Annotation Cluster**	3.374661
regulation of NK T cell differentiation	
positive regulation of NK T cell differentiation	

Supplementary Table S3. Two-proportion *z*-test of Rfam hits for miRNA and other ncRNAs. The proportion of the sum of the hits from Rfam matching to miRNAs divided by the sum of the hits from random intergenic regions matching to miRNAs vs the proportion of the sum of the hits from Rfam matching to all other non-coding RNA families. A one-sided two-proportion *z*-test was performed to examine if the proportion of miRNA hits was significantly greater. The *z*-scores were then converted to *p*-values.

Tissue	*p*-value
BrainE14.5	5.9×10^{-15}
Cerebellum	1.6×10^{-5}
Cortex	1.4×10^{-13}
Heart	4.9×10^{-3}
Kidney	7.5×10^{-1}
LimbE14.5	6.0×10^{-9}
Liver	3.1×10^{-2}
Lung	7.7×10^{-9}
Placenta	9.1×10^{-10}
SmIntestine	3.2×10^{-8}
Spleen	5.6×10^{-11}
Thymus	1.7×10^{-10}

Supplementary Table S4. List of microRNAs contained in regions of miR-like eRNAs with complimentary sites exclusively in the target promoter

Tissue	miRNA Family	Tissue	miRNA Family
BrainE14.5	[1]mir-484	Liver	[1]mir-10
	[1]mir-1224		[1]mir-193
	[2]mir-148		[1]mir-296*
	[3]mir-574		[3]mir-574*
Cerebellum	[1]mir-10*	Lung	[1]mir-877*
	[1]mir-149		[2]mir-574
	[1]mir-671	Placenta	[1]mir-8
	[1]mir-1224*		[1]mir-10
	[2]mir-574*		[1]mir-128
	[3]mir-320		[1]mir-145
	[3]mir-615		[1]mir-149*
Cortex	[1]mir-10		[1]mir-184
	[1]mir-320		[1]mir-193
	[1]mir-484		[1]mir-219
	[1]mir-574*		[1]mir-370
	[1]mir-877		[1]mir-877*
	[1]mir-1224		[1]mir-1224
Heart	[1]mir-10*		[2]mir-328
	[3]mir-574		[3]mir-122
Kidney	[1]mir-8		[3]mir-320
	[1]mir-149		[3]mir-484
	[3]mir-193		[3]mir-574
	[3]mir-296*		[3]mir-711
LimbE14.5	[1]mir-149	SmIntestine	[1]mir-32
	[1]mir-320*		[3]mir-574*
	[1]mir-551	Spleen	[1]mir-574
	[2]mir-10		[3]mir-320
	[2]mir-150		[3]mir-615
	[3]mir-432*	Thymus	[1]mir-10*
	[3]mir-574		[1]mir-320
	[3]mir-877*		[1]mir-328
	[3]mir-1224		[1]mir-672
[1]Hits from miRanda			[1]mir-877
[2]Hits from TargetScan			[2]mir-148
[3]Hits from miRanda and			[3]let-7
*Support from RNA-seq with Shorter Reads			[3]mir-574*
